# Supplementary material for: X-ray Structure Determination, Antioxidant Voltammetry Studies of Butein and 2′,4′-Dihydroxy-3,4-dimethoxychalcone. Computational Studies of 4 Structurally Related 2′,4′-diOH Chalcones to Examine Their Antimalarial Activity by Binding to Falcipain-2
Source: Molecules. 2021 Oct 28;26(21):6511. doi: 10.3390/molecules26216511 (PMC8588236; doi:10.3390/molecules26216511)
Supplement: Supplementary file 1 [file molecules-26-06511-s001.zip › molecules-1361607-supplementary.pdf]

Supplementary material

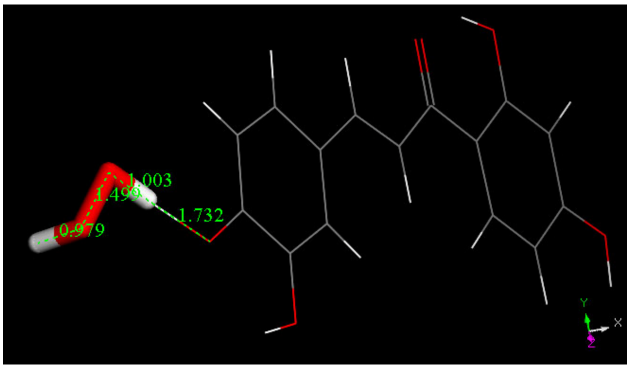

Figure S1. The structure shown in Figure 8 was approached by a proton, 2.60 Å apart from the O(superoxide) not engaged in H abstraction of Butein. After geometry optimization, formation of H_2O_2 (stick style) was obtained with further separation from the semiquinone Butein species, 1.732 Å, compared with 1.597 Å in Figure 8.

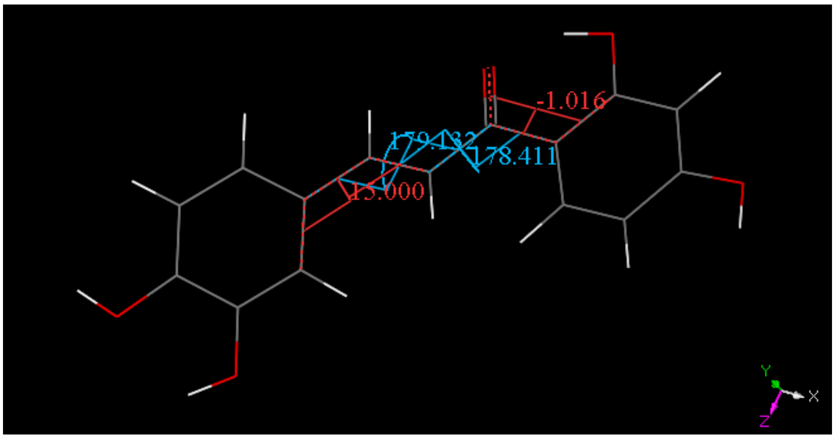

Figure S2. From DFT calculations, the Butein conformation is modified from the original catechol moiety torsion angle of 3.4° to 15°, and then a single point energy calculation is performed.

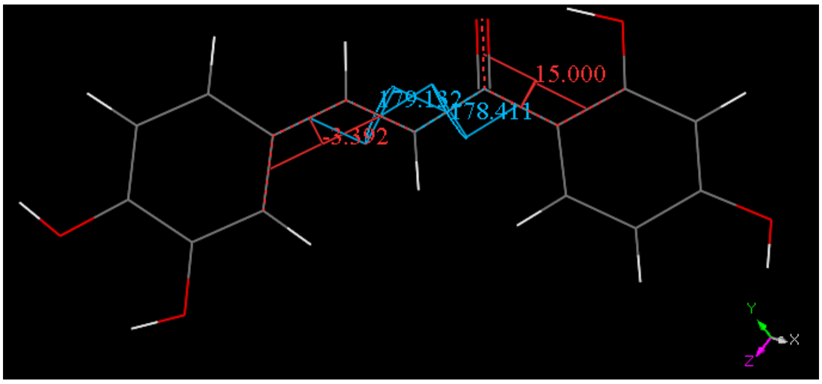

Figure S3. From DFT calculations, the Butein conformation is modified from the original non-catechol moiety torsion angle of -1.0° to 15°, and then a single point energy calculation is performed, see also Table 3.

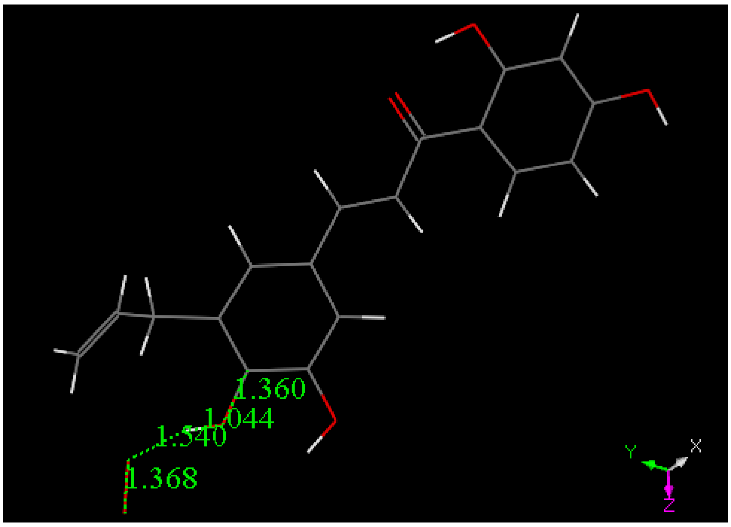

Figure S4. Geometry minimization of initially separated van der Waals 5-Prenylbutein and superoxide (2.60 Å) does not show capture of H4(hydroxyl) by the radical, with bond distance between O(superoxide) and H4 = 1.540 Å).

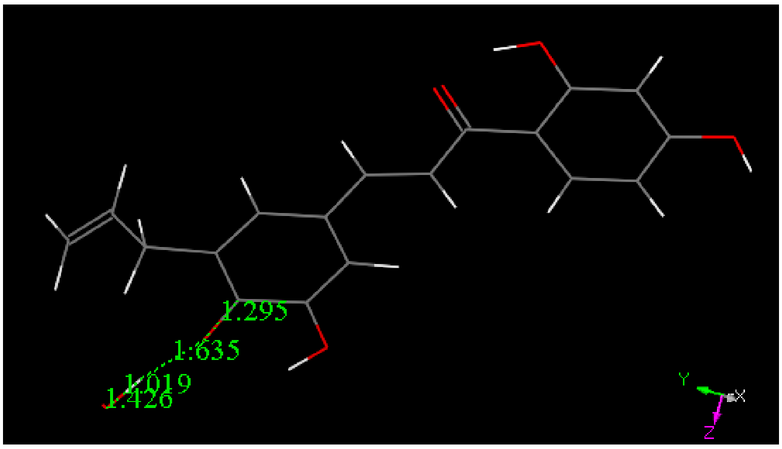

Figure S5. Geometry minimization of initially separated van der Waals 5-Prenylbutein semiquinone and O_2H (2.60 Å) converges to this minimum showing higher energy than that of Figure S4 (3.4 kcal/mol), suggesting no capture of superoxide by 5-Prenylbutein.

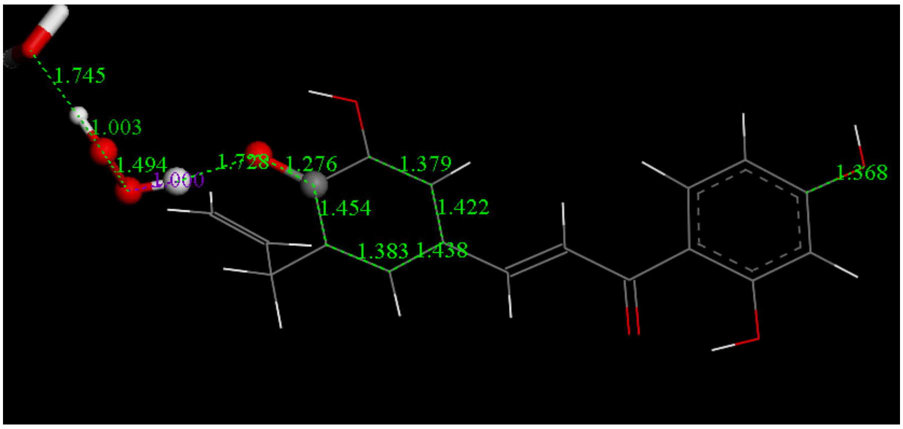

Figure S6. Geometry optimization obtained after placing $[H_3O]^+$ to the O_2H moiety of Figure S4. The system evolves towards $H_2$0---H_2O_2---5-Prenylbutein-semiquinone, demonstrating 5-Prenyl capability of scavenging superoxide (one H atom of water is hidden by its linked O(atom) in this view). In the semiquinone ring the C-O bond length of 1.276 Å has a double bond character, shorter than 1.368 Å single bond on the non-catechol ring (ring A right side of the drawing). In addition, the former catechol ring shows loss of aromatization, due to extended conjugation, as short C-C bonds (1.383 Å and 1.379 Å), alternate with longer C-C bonds (1.454Å, 1.422Å, and 1.478 Å).

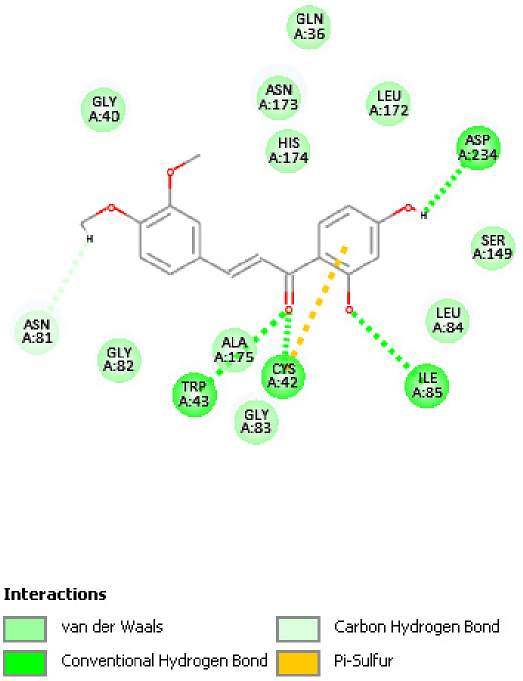

Figure S7. 2D interactions between DHDM **(2)** pose 2 and the active site of Falcipain-2, after calculating bonding energy.

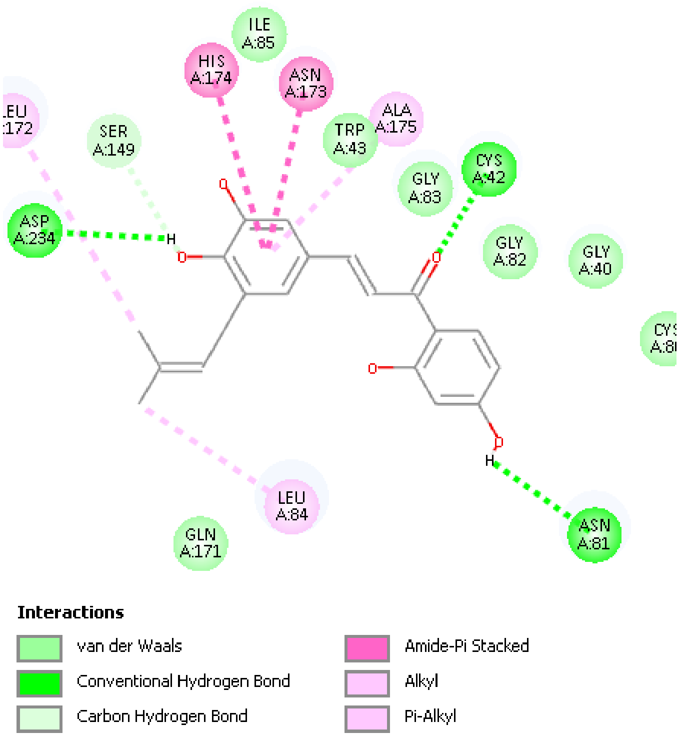

Figure S8. 2D interactions between docked pose 3 of 5-Prenylbutein and the active site of Falcipain-2.

Table S1. Comparison between bond distances in the crystal and DFT calculated Butein

Bond	Xray distance (ESD)	Calculated distance
O2-C4	1.375(2)	1.377
O1-C3	1.380(2)	1.381
O5-C13	1.360(2)	1.374
O3-C9	1.262(2)	1.280
C3-C4	1.395(2)	1.420
C1-C7	1.458(2)	1.457
C10-C15	1.404(2)	1.421
C10-C9	1.461(2)	1.476
C13-C14	1.395(2)	1.424
C3-C2	1.374(2)	1.391
C2-C1	1.403(2)	1.420

C1-C6	1.393(2)	1.417
C4-C5	1.382(2)	1.400
C10-C11	1.418(2)	1.440
C13-C12	1.380(2)	1.398
C7-C8	1.335(2)	1.364
C8-C9	1.460(3)	1.468
C14-C15	1.372(3)	1.389
C5-C6	1.387(2)	1.399
C11-C12	1.378(2)	1.403

DHDM

Bond	X-ray distance(ESD)	Calculated distance
O3-C3	1.3650(13)	1.375
O5-C9	1.2624(13)	1.280
O4-C17	1.4345(15)	1.479
C1-C2	1.4146(15)	1.424
C2-C3	1.3805(15)	1.392
C3-C4	1.4195(15)	1.434
C9-C10	1.4521(15)	1.475
C8-C7	1.3361(16)	1.365
C10-C15	1.4115(15)	1.420
C6-C5	1.3918(16)	1.401
C13-C12	1.3866(16)	1.398
C11-C12	1.3868(16)	1.403
O3-C16	1.4300(14)	1.456
O7-C13	1.3504(13)	1.374
O4-C4	1.3562(14)	1.368
O6-C11	1.3529(13)	1.360
C1-C6	1.3916(15)	1.411
C1-C7	1.4601(15)	1.453
C4-C5	1.3854(16)	1.402
C9-C8	1.4728(15)	1.468
C10-C11	1.4201(15)	1.439
C13-C14	1.4072(15)	1.413
